# Book Reviews

**Published:** 1820-04

**Authors:** 


### CRITICAL ANALYSIS

OF

### RECENT PUBLICATIONS, IN THE DIFFERENT BRANCHES OF MEDICINE AND SURGERY.

"I would have men know, that, though I reprehend the easie passing over of the causes of things, "by ascribing them to secret and hidden vertues and properties; (for this hath arrested and laid "asleepe all true enquiry and indications;) yet I doe not understand but that, in the practical "part of knowledge, much will be left to experience and probation, whereunto indication cannot "so fully reach: and this not only in *specie*, but in *individuo*. Yet it was well said, *Vere scire* "*esse per causas scire*."—BACON.

***Medical Report of the Fever Hospital and House of Recovery, Cork-street, Dublin, for the year ending 4th January, 1819.*** **By Richard Grattan, m.d. Fellow and Censor of the King's and Queen's College of Physicians, &c. 8vo. pp. 123. 1819.**

THE last accounts given in this Journal of the fever epidemical in Dublin, was produced from the report of Dr. Cheyne, in which the history of this malady was brought down to March 1818; we have now the continuation of it to a later period, in this report by Dr. Grattan, and in one, which will presently engage our attention, by Dr. John Crampton.

When the fever-hospitals were instituted in Dublin, it was expected that they would have been the means of arresting the progress of the epidemy; it seems, however, that they have done but little more than lessen its fatality; and, although its prevalence is now less extensive than it has been, this alleviation is to be attributed to other measures, of a political rather than of an expressly medical nature.

The fever is, it appears, propagated in a great measure by contagious influence; and Dublin has not, indeed, been totally free from contagious fever for many years past: but its late extensive prevalence has sprung from deeper sources, and it is to discussions on these, and on the means of obviating them, that a considerable part of the present work of Dr. Grattan is devoted. These subjects have become so well understood, since the accurate researches that have of late years been made respecting them by several excellent physicians, and by Malthus and other philosophical politicians, that it is not easy to advance any thing new respecting them. Dr. Grattan's views of the influence of moral as well as physical causes in giving rise to epidemic fever, coincide in most respects with those now generally taken of this subject; and his opinion, that fever originating from causes of an endemic nature, may at length become propagated from one person to another by contagious agency, seems to be too well established to merit further discussion. We therefore pass over in this general manner the subjects above alluded to, and some other observations relating to medical police, of merely local interest, to the author's observations on some points in the nature and treatment of fever: especially those respecting the physiological origin of this form of disease, and the use of blood-letting in its cure.

Dr. Grattan combats the general doctrine of the origin of fever from local disease of an inflammatory nature, especially as it is advanced by Dr. CLUTTERBUCK; but he errs, in including Dr. ARMSTRONG among those who support that doctrine. This physician decidedly opposes it, believing that fever does frequently exist without local inflammation being any-where present.

"The doctrine that *all* fevers are essentially inflammatory," Dr. Grattan says, "has not yet received sufficient confirmation, either from physiological reasoning, or from anatomical research, to satisfy us of its accuracy; and, until it shall be clearly proved that such is the case, those physicians who entertain a contrary opinion, and refer the immediate cause of fever to a disordered state of the nervous system, altogether distinct from inflammation, have at least an equal right to presume that their theory is correct." We must admit the conclusion, if the hypothesis that a disordered state of the nervous system is the immediate cause of fever, be as well supported as that which attributes it to inflammation. But is this the case? We shall not at present enter again into the discussion of this question; it is only the arguments of Dr. Grattan in favour of the doctrine he embraces, that will now become the subject of particular consideration.

"As far as anatomical investigation goes," the author remarks, "it would appear that, in many cases of death from fever, no traces of inflammation were observable after death, and if only a single instance of this kind could be advanced, it would be sufficient to overturn the opposite opinion. That such cases do occur, must appear evident from the observations of Dr. Macartney and Mr. Kirby, directed expressly to this subject, and published in the second volume of the Transactions of the Dublin College of Physicians. The opportunities which these gentlemen possess, and their acknowledged ability as anatomists, give to their testimony on this subject, and one so completely within their province, a degree of authority which cannot easily be questioned."

When those observations came under our notice on a former occasion, we endeavoured to show how far they are from furnishing any precise inferences like those the author here is inclined to deduce* from them: and we now find Dr. JOHN

* Those who are not acquainted with the observations here alluded to, should be informed, that they were made on bodies of persons *supposed* to die of fever, and which had been brought to dissecting-rooms after having been interred. We have daily occasion to remark, in making preparations of pieces of morbid anatomy, the disappearance of the indubitable signs of inflammation within a few days after

CRAMPTON expressing similar opinions; as will be presently shewn. Besides the remarks here alluded to, it must be understood, that Dr. MACARTNEY's decisions rest on a gratuitous distinction he has made between certain appearances and those of "real and pure" inflammation; and such a distinction as, we believe, will be by no means generally admitted.

Dr. Grattan then argues against the inference, that a distended state of the blood-vessels after death, is a proof of their having been in the same state during life, and of the existence of inflammation. But it is not on such observations and such inferences as these, that the doctrine he opposes is founded; nor on the loose analogies which he with equal propriety exposes in the following passage:

"I have seen instances of patients who, a few hours previous to their death, exhibited marks of apparent inflammation, tending rapidly to gangrene, and which state clearly depended on a deficiency of nervous energy, and constituted, in fact, the commencing dissolution of the part. This occurring on the surface of the body, is evident to the sight, and cannot be questioned. May not then the same process equally take place in the brain; and thus, an appearance which is only the effect, come to be considered the cause of the disease? This is, I think, extremely probable, and it is likely, that in this way very erroneous opinions may be adopted by the mere anatomist, with respect to the immediate cause of fever."

We do not blame the author for not having fairly met the question: we well perceive, that the nature and object of his report did not admit of a precise discussion of even the principal points of it; but we think he would have done better, had he confined himself to a simple exposition of his own reasons for adopting the opinions he has formed on the subject under consideration, rather than notice it in the partial manner he has done on this occasion. Dr. Grattan's arguments lead him to the conclusion, that "the doctrine which teaches that fever is a disease of the nervous system, and at the same time admits that this diseased action does often occasion local inflammation, seems to be nearer to the truth, than if we were to ascribe fever to either of those causes exclusively. It embraces both theories; and, in a practical point of view, comprehends every case, and every variety of treatment, which can be employed in the management of fever." This is not quite a correct view of the hypothesis the author has opposed: because that teaches, not that inflammation is an accident or contingent in fever, but that it is the *cause* of the disease; and therefore, we do not see how the hypothesis of Dr. Grattan can be said to embrace this also. We

---

death. These considerations alone are sufficient to invalidate the induction above designated.

turn to Dr. Grattan's observations on the use of blood-letting; and the gratification we experienced in the contemplation of this part of his report, will lead us to consider them in a somewhat comprehensive manner: though, as he himself inculcates, we acknowledge their application only to the peculiar circumstances to which he expressly applies them. Dr. Grattan proves, in the most satisfactory manner, that copious blood-letting is not necessary, in order to lead to a favourable termination, in the generality of cases, the fever at present epidemical in Dublin; and his practice appears to have been not less successful than that of Drs. Cheyne and Percival under similar circumstances, although he employed this remedy in a somewhat more sparing manner than the physicians just named. In the more simple cases, only purgatives and diluents were prescribed by Dr. Grattan; but, in those with complicated symptoms, blood-letting was generally resorted to. The number of cases of the latter kind which occurred to his observation in one year was 544, of which only 32 proved fatal; which is only one in seventeen: and this, in cases which may be considered to have been of the more severe kind, is certainly a low ratio. Of this number 316 had pectoral symptoms, for which 116 were blooded from the arm, the total quantity taken being 634 ounces; or, on an average, five and a half from each patient. In general, the quantity directed to be drawn varied from four to six ounces; the same patient was seldom bled more than once, and very rarely more than three times. In 268 patients the head was affected, and 98 were blooded from the temporal artery, the total number of ounces amounting to 508; giving an average of about five ounces from each patient.

It is remarkable, that no instances in which *gastric* disorder was particularly severe, are mentioned amongst the above complicated cases. We have generally found such a complication to be one of the most frequent and most distressing forms of disease, in epidemic contagious fevers in general; and the last report of Dr. Cheyne shows it to have been very common during the period that report embraces.

In order to show more clearly the effects of moderate blood-letting, Dr. Grattan gives an account of all the deaths (which, it appears, were only the thirty-two above designated) that occurred during the year amongst the patients in the wards under his direction. These details lead to inferences of the most absolute nature in favour of Dr. Grattan's general practice. We can only suppose, that the severe complication under consideration might probably have been prevented, in a few instances, had early blood-letting been resorted to: but this remark does not apply to Dr. Grattan's practice, because the few cases here designated did not come under the care of the able physician here alluded to, until the disease had, apparently, already made too great progress to be benefitted by such means. The reader must however bear in mind, that a very great portion of the patients received into the hospital, had been subjected to debilitating causes previously to the attack of the disease: such as want of sufficient nutriment, and depressing moral influence. Our own observations have led us to believe, that the lower class of Irish peasantry and labouring artisans, under better social circumstances, neither bear nor require such copious blood-letting as those of England, Scotland, and some other nations of Europe, in diseases of the most purely inflammatory nature,—as pleurisy; a diversity that may be satisfactorily accounted for, by the differences of climate, and of the diet and general habits of the individuals.

We meet with many observations of considerable value on the use of several other remedies in the cure of fever, in the Report before us: by adducing some of these, we shall furnish our readers with useful practical information, and at the same time give them an idea of Dr. Grattan's very judicious mode of practice.

We shall first notice a remark on a sort of delirium, in which the patient, though evidently labouring under disease, contends that he is quite well, or affirms that he has slept, although we know he has had no sleep. This apparent want of consciousness, this insensibility to their own feelings and internal sensations, is always alarming, and should render the physician more assiduous in his inquiries, and more guarded in his prognosis. We have found patients in this state say at first they have no head-ache; but, on being made to shake their head, they often then complain of it. Dr. Grattan says,

"The particular condition of the system on which this state depends, I believe, is not always confined to the brain alone. I remember to have seen a patient who, having through some accident received a blow on the chest, was seized with slight febrile symptoms, and was said to talk at times incoherently. He was dressed and walking about, complained neither of his head nor of his chest, and there, in fact, appeared no symptom to indicate immediate danger. His friends wished to have him removed to the hospital, and an order was given for his admission. He was not removed; and, on enquiring the next day, I was surprised to learn that he had died in about an hour after I had seen him."

In the low delirium, unattended with pain of the head or fulness at the temples, and when the heat of the forehead is not increased, and the face is pale, and the features hollow and contracted, Dr. Grattan has not found blood-letting beneficial. In many such instances, he has witnessed the recovery of patients in such a state, under a treatment very different from the depleting system. Wine, and other stimulants, moderately given, camphor, barm, muriatic acid copiously diluted, gentle aperients, successive blisters, and calomel, have often apparently rescued the patient from impending death. We frequently find a similar series of symptoms come on at the end of a few days, or a week or two after the receipt of severe wounds, especially from gun-shot, and these are commonly treated by judicious practitioners by similar means: bleeding here is decidedly injurious. A full dose of opium is often productive of a degree of benefit that will surprise those not aware of its efficacy. However, Dr. Grattan properly remarks, it will sometimes happen that, when stimulants are required to support the general tone of the system, moderate topical blood-letting may be beneficially employed, and be even necessary, to relieve local congestion. When the head is affected, Dr. Grattan has always found that more decided and immediate relief was obtained by taking five or six ounces of blood from the temporal artery, than by abstraction of a much larger quantity from the arm. In cases of the kind above designated, Dr. Grattan's observations have led him to place much confidence in the efficacy of mercury. In most instances, he says, "the moment the mouth is rendered sore, the symptoms become less violent, and the patient thenceforth gradually recovers." In many cases, the mineral acids and barm are highly beneficial, especially in the latter stages of fever, in persons of debilitated habits, when the tongue is rough, dry, and black, and the teeth covered by a brown and adhesive crust. To the testimony of Dr. Grattan in favour of the muriatic acid in such cases, we may add, that two drachms of muriatic acid in a quart of water, to be used as a drink after short intervals, is a common prescription of Dr. Armstrong, under similar circumstances, for the patients at the Fever Institution in London.

---

***Medical Report of the Fever Department in Steevens's Hospital, containing a brief Account of the late Epidemic in Dublin, from September* 1817, *to August* 1819. By John Crampton, m.d. Physician to Steevens's Hospital, &c. 8vo. pp. 63. Hodges and M'Arthur, Dublin, 1819.**

Dr. Crampton says it had been his intention to have abstained from giving any account of the late epidemy. "So many fever reports have been published, I considered the subject as nearly exhausted: in almost every remark I could make, I found myself anticipated." We feel some degree of surprise, and much disappointment and regret, on finding such observations made by the author of the "*Clinical Report on Dropsies*," inserted in the second volume of the Transactions of the Dublin College of Physicians. We looked with confidence to this physician, for some important addition to our knowledge respecting the relations of morbid appearances on dissection to the phenomena of fever during life; and we cannot but think, that he could have produced it. The researches of Drs. Cheyne, Percival, and Mills, have disclosed much on this subject, in regard to the epidemy of Dublin; but something yet remains to be effected in this very important point of pathology. Dr. Crampton thus continues: "I feel it however due to the profession, and due to the government of the country, who have so liberally contributed to relieve the poor suffering from distress and disease, and who have interposed their arm, and saved a populous nation from pestilence, to detail the arrangements that were made in Steevens's Hospital, and explain the treatment which was employed with a number of patients amounting nearly to five thousand."

Dr. Crampton has however adduced some general remarks on the nature of the morbid appearances after death, which we shall wholly transcribe; but he has not indicated the relations of these to the symptoms in fever in a particular manner. It is the want of this that we so much regret. He says,

"On the subject of the morbid appearances found in those who die of fever, all those who are hospital physicians, and who have either practised or superintended dissections, are fully agreed. A few of those who have not seen bodies opened themselves, but who rely on the opinions of others, founded on the examination of such subjects as are generally brought to the dissecting-room, without knowing the previous history of the disease, have taken a different view of the question.

"I witnessed myself most of the examinations which took place at the hospitals of the House of Industry, not only of those who died in the Hardwicke and Whitworth hospitals, but those who fell victims to fever in the Richmond Penitentiary, which at that period accommodated about 500 patients.

"The appearances which I observed in the brain and its membranes, were fulness and distention of the vessels, the arteries as well as the veins, the former often appearing as if subjected to an anatomical injection; extravasation of blood was seen in a few instances, sometimes under the arachnoid, and again at the base of the brain; effusion into the ventricles and under the arachnoid was frequently witnessed, as well as into the spinal canal; a thickening and opacity of the arachnoid and of the pia mater was not an unusual occurrence, and lymph was often thrown out, sometimes a wheyish fluid, and again a gelatinous substance. These membranes occasionally manifested more unequivocal signs of previous inflammation, exhibiting their surfaces superficially ulcerated and covered with a purulent discharge. Some other varieties in the appearances found in the brain were observed, but they were mostly of the same character.

"In this account of the morbid changes in the brain in fever, I find I am supported by Drs. Percival, Cheyne, Mills, Bateman, and Duncan, in their respective accounts of the late epidemic; similar views are also taken by the French writers on fever, in the "Dictionnaire de Sciences Medicales," supported by the dissections of Baron Larrey, and other French anatomists.

"The authorities of Dr. Macartney and Mr. Kirby are adduced by Drs. Barker* and Stoker,† in their respective Fever Reports, to support a different opinion. The subjects‡ which these distinguished anatomists examined, were brought for dissection in both instances to an anatomical theatre; their previous history was not always known further than that they were supposed to be cases of fever, because spots were observed on the skin. This alone, however, must be allowed to be a very equivocal foundation to establish that they were fever cases; it is not always easy in a living subject to discriminate between febrile petechiæ and other eruptive appearances on the skin; the distinction, I believe, is, at least, equally difficult in the dead body.

"In the fever reports just cited, congestion in the venous system of the brain more especially, is stated to be present in fever; and it is added, that, as the appearances which are usually the result of inflammation were not observed in the subjects thus examined, 'the congestions observed after typhus fever differ from those of genuine inflammation.'§

"It may be observed, that this kind of venous congestion readily takes place in any subject, if, during its removal, the body is not kept at least in a horizontal position; and, if the head be allowed to hang in a depending posture, fullness of the veins of the head is immediately induced. If this circumstance be coupled with the doubt which must remain as to the actual disease of which the subjects which were thus examined died, I apprehend no inference should be drawn as to the condition of the vessels of the brain in fever, from examinations made under those circumstances.

"I regret that I am obliged thus to differ from those intelligent friends of mine for whose opinions I entertain the highest respect. The decision on this difference of opinion, however, must in fact depend on the rigid test of experimental inquiry, and the point be determined, not by dissections, where the slightest doubt remains whether they were fever cases, but by examinations made on a large scale, where all the circumstances are known from the beginning. Indeed, this has already been done to a considerable extent by Dr. Cheyne, in his Second Medical Report of the Hardwicke Fever Hospital.

"Where the organs in the cavity of the chest were most pressed during fever, the fatal cases almost invariably exhibited changes of structure, which all anatomists allow to be the result of inflammation;

---

* *Trans. Medical Association, Coll. Physic. in Ireland,* vol. ii. p. 574.

† Ibid. p. 431.

‡ "A great proportion of the subjects for anatomical lectures in Trinity College, during last winter, appeared to have been of the present epidemic, as they had petechiæ on the surface." Ibid. p. 574.

§ *Dr. Barker's Report.*

recent adhesions of the *pleura*, exsudation of wheyish or sero-purulent fluids, effusions of lymph and formation of false membranes, besides analogous changes of structure in the *mucous*, *bronchial*, and other textures. Tubercles were often found in the lungs in every stage; nor did the heart and its membranes escape: in a man who died in the Whitworth hospital of a severe relapse, the pericardium was found full of a wheyish fluid, and the heart* covered with a recently-formed coating of lymph.

"In a considerable number of those examined, the liver was found tuberculated and diseased in a variety of forms; but this is not surprising, when we consider the number of intemperate drunkards with worn-out viscera who occupy that great depot of poverty, the House of Industry, and who, when afflicted with febrile symptoms, are from thence transferred to the fever-wards. The diseased state of these organs could not with propriety be regarded always as the only cause of fever, although it was certainly instrumental in hastening the death of the patient. In many instances, however, the serous membranes covering the liver were found with fresh coatings of coagulable lymph, serous effusions, and recent adhesions were also observed; and these appearances could be connected with severe local symptoms, and other symptoms of inflammation, which must have existed shortly antecedent to death.

"In the gastric form of fever, at least in the severe and fatal cases, the *mucous* coat of the stomach, and in some instances that of some portions of the intestines, was found after death slightly inflamed. If the symptoms which denoted these occurrences were neglected, the *serous* coats and the *peritoneum* soon became involved; numerous instances of such appearances are on record in our hospital journals, and portions of the morbid parts are preserved, which illustrate these appearances.

"In those who died of the various forms of dysentery combined with fever, it is scarcely necessary to say, that inflammation was present at some stage of the disease, and that erosion and ulceration were the result. On the subject of a tuberculated or warty appearance of the villous coats of the intestines, which was another form of deranged structure occasionally met with, especially with children, I have already offered an opinion in another† place, so that I shall not repeat it here. Those however who wish for authentic information on the diseased appearances to be found in those who die of fever, will find it in a tabular form, in Dr. Cheyne's‡ Second Report of the Hardwicke Fever Hospital. There are also some fever dissections given by Baron Larrey, in that interesting volume in which he describes the fever which occurred amongst the French troops on their retreat from Moscow, and by Broussais.§ But the anatomical preparations made at the House of Industry, form an incontestible chain of evi-

* This heart is preserved.

† *Dublin Hospital Reports*, vol. ii. p. 286.

‡ Ibid. p. 1.

§ *Histoire de Phlegmasies Chroniques.*

dence, which ought to convince the most sceptical that, in the severe forms of the fatal cases of fever, there is something more than *venous congestion* to be attended to."

We are led to hope, however, that Dr. Crampton has not thus given up this matter, by his observation that "the subject is still under investigation, and probably may come before the public at some future period, after a greater mass of authentic evidence has been collected."

On the arrangement of fever-hospitals, and the treatment of the malady to which it relates, this report contains much matter of rare value, which we shall not attempt to display in an abstract of what is already detailed with the utmost conciseness; but earnestly entreat our readers to peruse in the original. They will, indeed, be readily induced to make themselves acquainted with the method of treating fever patients adopted in an hospital, where the ratio of mortality ranged from one in thirty-five to one in sixty-nine. This evidence is the best testimony of the excellence of the practice resorted to; and we shall not be the means of rendering it less generally beneficial, by giving any partial, and consequently imperfect, account of the measures it embraces.

---

*Medico-Chirurgical Transactions.* Vol. X. Part I. London, 1819. Longman and Co.

[*Continued from page* 141.]

*History of a Case of Nephritis Calculosa, in which the various Periods and Symptoms of the Disease are strikingly illustrated, and an Account of the Operation of Lithotomy, given by the Patient himself.* By Alex. Marcet, M.D. F.R.S. one of the Vice-presidents of the Medico-Chirurgical Society.

This paper presents several interesting circumstances, chiefly in the illustrations of the different periods of the disease noticed in the title, and in the manner in which the intelligent patient related his own sufferings: yet we must not indulge in an account of them, to the exclusion of matter of more immediately practical utility.

*Case of a Periodical Affection of the Eyes and Chest.* By John Bostock, M.D. F.R.S. & L.S.

The learned author of this paper has not expressed his own opinions respecting the nature of the disease, and its relations, of which the history is here adduced; and we have not been able to form any conclusions on those points, nor any particularly useful practical deductions from it; we must, therefore, be permitted to pass it over.

*A Case of Chronic Inflammation of the Larynx, in which Laryngotomy and Mercury were successfully employed.* By MARSHALL HALL, M.D. of Nottingham. Communicated by Dr. ROGET.

THE observations related in this paper possess a remarkable degree of interest; and, with the accumulating cases of a similar kind, will probably render laryngotomy and bronchotomy familiar operations. A careful examination of a great proportion of the recorded fatal cases of inflammation of the larynx, will show that death has commonly resulted from suffocation, induced by functional constriction of the glottis, rather than from obliteration of the canal by effused and coagulated lymph. This would, it is highly probable, have been the case with Dr. Hall's patient, had not the operation been timely resorted to.

The patient was a woman aged fifty-three: she had for two months been affected with hoarseness, a hard dry cough, which increased in severity during that time, and then became attended with a sense of "tightness in the throat," and a symptom which would probably have been unnoticed by the generality of practitioners, but which did not escape the author of the essay on *Diagnosis:* the patient said she was unable to "snuff up" through the nose in the ordinary way. This Dr. Hall supposes to be "an effect of the partial closure of the larynx; for, to produce this snuffing, it is necessary that a certain *quantity* of air should be drawn through the nostrils with a certain *velocity;* and, in the present instance, the *quantity* of air admitted appears to have been too small." This explanation appears satisfactory; and we think the observation a very useful addition to semiology. There was at this time, also, some degree of difficulty of breathing. These symptoms increased for two months longer, when she began to experience, in addition, a degree of difficulty in swallowing. After another month, at the beginning of March, "she observed a swelling, rather diffused, about the size of a pigeon's egg, over the upper part of the thyroid cartilages, with an increase of the dyspnœa and dysphagia. A liniment was employed for this tumor, by which it was reduced in size, and the difficulty in breathing and in swallowing was diminished. In a short time, however, these symptoms became again aggravated, and they continued to increase until the month of August." At this time she consulted Dr. Hall.

"She was then affected with a degree of hoarseness, which rendered the voice scarcely audible. There was a perpetual dyspnœa referred by the patient, by the noise in breathing, and by the sound of the cough, to the upper part of the larynx. She swallowed with great difficulty and effort. There was an obvious general tumefaction of the parts about the larynx, occupying the left rather more than the right side. She stated that she experienced great difficulty in walking up a hill or pair of stairs."

The cough had recently been attended with the expectoration of viscid mucus, once tinged with blood.

Dr. Hall recommended "five grains of the pil. hydrarg. to be taken every night and morning, half an ounce of the sulphat of magnesia twice in the week, four leeches to be applied over the larynx every other day, and a lotion constantly, when the leech-bites were not bleeding."

"On the 22d of August," Dr. Hall continues to observe, "I again saw Mrs. H. The symptoms were unabated. The mercurial had produced no effect on the gums. Mrs. H. was now induced to remain a short time in Nottingham. There was a degree of emaciation and debility; the pulse was rather frequent and feeble; the appetite impaired. The pil. hydrarg. was continued three times a-day.

"On the evening of the 24th, Mrs. H. was seized with an alarming fit of dyspnœa, to which I was witness. There was the greatest anxiety of countenance and manner; and, in the breathing, every auxiliary muscle of respiration was called into exertion, and there was every appearance of impending suffocation. The dyspnœa had abated somewhat in violence, and there had been similar fits of dyspnœa before, or I should have immediately recommended the operation of laryngotomy. The difficulty of breathing abated gradually, and I left my patient in her usual state of dyspnœa.

"In consultation with Mr. Oldknow, a most skilful surgeon of this town, it was concluded that the operation of laryngotomy was necessary, to avert the danger of suffocation incurred during the fits of dyspnœa."

Mr. Oldknow performed the operation by making

"An incision through the integuments, beginning a little above the thyroid, and terminating a little below the cricoid cartilages. The external surfaces of these cartilages were then exposed, partly by incision and partly by the finger. An opening was then made into the larynx a little below the most prominent part of the thyroid cartilage, and extended downwards to the upper margin of the cricoid cartilage. This incision not being sufficient for respiration, a crucial incision was next made through the membranous connection of the two cartilages; but still with an unsatisfactory result, for the breathing was performed only in part, and with great difficulty, through the aperture thus made. A circular portion, about $\frac{1}{8}$ of an inch in diameter, was therefore removed from the lower and lateral part of the thyroid cartilage, when the respiration became perfectly free, and the patient experienced the greatest possible relief. The cut edges of the integuments were kept asunder by straps of sticking plaster, passed from them towards the nape of the neck."

This able surgeon makes some remarks on the propriety of the mode in which he performed the operation in this case, the validity of which must be acknowledged.

"This operation (Dr. Hall then states) afforded immediate relief to the respiration, and Mrs. H. slept soundly through the ensuing night, for the first time for a long period. Deglutition continued difficult, and always induced coughing during the five or six subsequent days. The cough raised some viscid mucus, which was forced through the orifice made by the operation. The voice was quite lost.

"On the day of the operation the pil. hydrarg. was omitted, and the ung. hydrarg. was prescribed to be rubbed in, in the quantity of half a drachm morning and evening. The ol. ricini was ordered to open the bowels.

"On the 28th the mouth became sore. Mrs. H. soon afterwards experienced a mitigation of the difficulty in swallowing; and, on applying the finger to the opening into the larynx, she found, in a short time, that the tightness in the respiration was also diminished, and that she could breathe with greater facility than before the operation; and, as she expresses it, more freely through the nose."

The amendment continued progressive. The orifice in the larynx had closed on the 11th of September. Soon after this time, however, the patient left her bed-room, and exposed herself to the currents of air in a room with three doors. A return of difficulty of deglutition, and a loss of the voice, which had been regained, was the consequence; but, as soon as friction with mercurial ointment produced ptyalism, the difficulty in swallowing began to disappear, and the voice became again improved. On the 27th of October, it is stated, that she only suffered from the effects of the mercury on the mouth. On the 8th of the ensuing January, Dr. Hall heard the most satisfactory account of the patient's recovery and general health.

We have been led to give so detailed an account of this case, from its showing, in a very clear and forcible manner, how much danger is dependant on functional (or *spasmodic*, as it is ordinarily termed) contraction of the glottis in diseases of the larynx; another instance of the safety with which the operation of laryngotomy may be performed, even under apparently unfavourable circumstances, and the remarkable influence of the effects of mercury in relieving chronic inflammation of the part just alluded to. The treatment adopted, Dr. Hall remarks, is due almost exclusively to the suggestions of living authors; especially to Baillie, Farre, Lawrence, Charles Bell, &c.

*Observations upon the Morbid Appearances and Structure of Bones; being the Sequel of a former Paper.* By John Howship, Esq.

This paper is produced, as a continuation of that in the eighth volume of these Transactions, by the same author. Mr. How-

SHIP's observations on the present occasion relate to spina ventosa; and enlargement of bone with increased interstitial deposit of ossific matter, producing a more dense and compact structure than natural; as happens in what he calls *healthy ossific inflammation*.

Mr. Howship considers spina ventosa to be an enlargement of the bones, including the whole diameter of the affected part. This he supposes to arise, in the first stage of the disease, from an increase in the natural secretions, depending on excitement of the fine secreting membranes, which generally commences in those membranes within the great channel or medullary cavity of the bone, and afterwards extends throughout the whole of the membranes to the outside of the bone. The pressure of the fluid thus effused acting as an expansive force on the bone, unfolds its texture, in a manner, and dilates it in the way we commonly observe in this disease." This hypothesis is plausible to the imagination; but Mr. Howship acknowledges that he has "not actually examined the recent soft contents of any early specimen of this disease." He has formed it from reasoning analogically from the opportunities he says he has had "of tracing the natural secretions in the cavities of bone under various states of disease, some of them producing almost precisely thes ame effects as take place in spina ventosa." Mr. Howship, we are sure, has too much zeal in the cause of pathology, to suffer himself to rest with confidence on a doctrine raised on such a foundation, and too much industry to permit him to neglect any means in his power, which may tend either to confirm or to refute it. Amongst his citations on this subject, there are two cases from DUVERNEY, in which the appearances correspond nearly with the above description; and the drawings he has made from some preparations, display the sort of dilation he has designated. We recommend this paper wholly to the perusal of our readers: the matter of it cannot be either well abridged, or understood, without the aid of the specimens of Mr. Howship's eminent talents in delineation with which it is accompanied. We must however add to the above observations respecting spina ventosa, that Mr. Howship thinks the "fluid secretions within bones affected by this disease have the appearances that are known to belong to scrofula, and seem to be principally injurious by the expansion they produce;" though, at length, the membranes themselves become thickened in proportion to the degree of extension they undergo; and in some parts they are occasionally found to have become cartilaginous, and even ossified. Sometimes, too, suppuration takes place in the bone, and various sinuses are produced, opening on the external surface of the bone.

*Case of Carotid Aneurism.* By J. P. VINCENT, Esq. Surgeon to St. Bartholomew's Hospital.

THE patient, a man aged fifty-two, about ten weeks after "he had been knocked down, and beaten about the right side of his head, in which he had suffered much pain," discovered a pulsating tumor behind the angle of the jaw on the side on which he had received the injury. Seventeen days after this, on his admission into St. Bartholomew's Hospital, the tumor was about the size of a pullet's egg. He was suffering severe pain over the right side of his head, and the whole arterial system beat with powerful action. He had been taking mercury, and was in a state of ptyalism. On account of this last circumstance, the operation was deferred nine days, during which interval the tumor had somewhat subsided.

"In reference to the operation, Mr. V. observes, it is proper to notice, that the omo-hyoidæus muscle so completely traversed the wound, and its connections being more than ordinarily firm, it could not be pressed aside, and some of its fibres were necessarily divided. The artery was brought into view on the inner side, next the trachea; and the theca was so firm and dense, that it was necessary to use the knife in separating it; after which a single ligature was drawn upon it. The internal jugular vein did not at all protrude in the way.

"After the operation the right subclavian artery beat with increased force; but this did not continue. The pulsation in the aneurismal tumor did not cease on drawing the ligature, although it was much more faint. The patient said he had lost the severe pain in his head, and felt only a slight pain over the right eye, and an uneasiness in the right shoulder, which soon went off. He suffered no irritation in the larynx or pharynx. The pulse at the wrist was much weaker than before the operation."

The only inconvenience experienced in the evening after the operation, was a slight pain about the forehead, and also in the abdomen. The pulse was eighty, and soft. The tumor was reduced in size about one half, and was faintly beating. The following day the patient had no pain in the head, nor any other unpleasant symptom, than "much uneasiness and sense of feeling in the abdomen, extending from the epigastric to the right hypochondriac region."

On the ensuing day, the tumor was not more than a quarter of its size before the operation, and there was no pulsation in it. The ligature did not come away until the twenty-second day from the operation, when the patient immediately lost all uneasiness in the abdomen, which had never before ceased since the operation. Ten days after this, the report is, that

"The right side of the neck is very tumid; the swelling extending from the middle of the wound to the lower jaw, affecting deglutition, but not at all the breathing. The fungous granulations are glassy, and the wound produces no discharge. The tongue is covered with a white coat; his pulse is quick, and he has had a disturbed night; the swelling however did not increase. About eight o'clock in the evening he became very ill, complaining of being low and uneasy; had great difficulty of deglutition; a fit of coughing came on, and respiration grew difficult. He was quite sensible. Mr. Lawrence, happening to be at the hospital at this time, saw him, and made an incision into the aneurismal tumor, from which a small quantity of pus and coagulum issued: he expired immediately afterwards."

The interesting nature of this case induces us to give in detail the account of the appearances on dissection, and Mr. Vincent's remarks on the causes of the curious phenomena which were observed.

"An examination of the state of the parts was made fifteen hours after death. The process of obliteration of the artery below the ligature was complete: the vessel being perfectly closed, a plug was formed, extending downwards as far as the division of the arteria innominata. The artery above the ligature was open and inflamed, and pus was found in it. The aneurismal sac had undergone the proper changes to effect a cure; it was contracted around a firm coagulum, and the inner surface of it bore no indication of having been inflamed. Thus the pus, which issued from the incision, must have been the product of the inflamed artery. The sac was formed at the bifurcation of the common carotid. The aorta and other large arteries were so increased in size, that the area of their sections was nearly doubled. Globules of air were found adhering to the inner surface of the aorta and other large arteries; and in the head air was found under the tunica arachnoidæa. The bulk of the swelling in the neck, which came on the day previous to his death, seemed to be formed of serous effusion in the cellular tissue.

"In viewing the progress of this case, it seemed to be a striking effect of the operation, to subdue the action of the arterial system; as, from the time it was finished, the pulse became so very much softer than it had been before. And the uneasiness and feeling of fulness of the abdomen may be fairly attributable to the presence of the ligature so near the par vagum, keeping up irritation upon it, as the whole sensation ceased the moment the ligature was removed. It is probably to the diseased state of the vessel that the inflammation of that portion of the artery which was between the ligature and sac must be referred: and, perhaps, in the fact of air being found introduced into the arterial system, may be discovered the cause of the sudden termination of life."

*The Use of Arsenic in the Cure of Chorea.* By Mr. Salter, Surgeon, of Poole. Communicated by Mr. Travers.

Two of the cases here related had been treated, without success, by the purgative plan. In two others, the arsenical solution was resorted to in the first instance: in the whole the remedy was effectual.

*On a new Method of preparing Pharmaceutical Extracts.* By John T. Barry. Communicated by Dr. Marcet.

The object of this paper is to make known to medical practitioners a mode, invented by Mr. Barry, of evaporating vegetable juices into the form of extracts, in a vacuum and by the heat of a water-bath; by which empyreuma, and the influence of the air on the substance during that process, are prevented. The properties of the preparation thus made, are consequently more constantly the same, and at the same time more powerful in a given bulk, than when the ordinary method is adopted. As Mr. Barry has obtained a patent for his apparatus, it would be useless for us to give a description of it in this Journal, supposing even that any of our readers might be themselves disposed to make use of it.

"With respect to the strength of extracts made in vacuo," says Mr. Barry, "I have not yet gained sufficient information to be able to present a view of the relative proportions which they bear to the common extracts; but I have been informed by several medical friends who have given them a trial, that they find them materially stronger. Perhaps some gentlemen will consider the subject sufficiently deserving of investigation, to collect such a statement of cases as will enable them to present to the Society the relative doses. I shall be glad to offer for the acceptance of such, specimens of any kind which they may be inclined to make use of."

This seems to be a very useful pharmaceutical improvement, for the variable qualities of the extracts ordinarily prepared, has been a subject of complaint by most observant medical practitioners.

---

*On the Malformations and Diseases of the Head.* Illustrated with Etchings. By William Wadd, Esq. f.l.s. Surgeon-Extraordinary to his Royal Highness the Prince Regent, &c. &c. 4to. pp. 21. Callow, London; 1819.

This fasciculus is intended to form a continuation of the former valuable works of the author, in which he has illustrated many interesting facts in pathology, by connecting graphical delineations of morbid alterations of structure with remarks on the most important circumstances respecting their nature and the mode of cure. The present collection will, however, prove interesting to persons not possessed of the former volumes of "*Cases in Surgery*," from the subjects of it being of so distinct a character.

We are not disposed to attribute much value to graphical representations of morbid structure, in the generality of subjects, and as they are commonly executed; but this is not the case with those of Mr. Wadd: he has chosen such facts as have occurred to his observation as may be thus usefully illustrated; and, whilst reflecting on them, he has taken up his pencil or etching-needle, and thus added force and precision to his verbal descriptions. No one but a pathologist can give appropriate accuracy and clearness to the more minute and essential points of graphic representations of subjects of this kind, and many of those of Mr. Wadd equal, with respect to execution, almost any specimens of etching we ever witnessed. Talents of this kind applied to pathology serve really to illustrate it.

The present fasciculus comprises descriptions of a remarkable formation of the cranium of a person who had for many years been in an insane state of mind; a representation of the head of a maniac, and of a distinct section of his skull, where great distortion was produced by a deposit of bony matter between the tables of the bone; and of two other cases, where a morbid state of the cranium from external injury had produced insanity: the portrait of a man whose head was crushed against a wall by a cart-wheel, by which accident a portion of the temporal bone was driven into the brain, a part of which appeared on the surface of the integuments, and the parietal bone also fractured, followed by exfoliation of a considerable portion of it. The man recovered from this accident, and lived twenty years, without any alteration of his corporeal functions or mental faculties, continuing in laborious exercises to the day of his death. The ninth plate represents a case of cancer of the eye-lids, originating from a violent blow, from which inflammation and sloughing of the eye ensued in the first instance. The disease had affected the brain previously to the patient's death. The representation of the face and upper part of the body of an anencephalous fetus. This instance shows, like all other cases of this kind, a preternaturally-large development of those parts of the head which remain. The last plate is of a curious head from Alpica, in the possession of Mr. Carpue; and, Mr. Wadd says, Dr. Leach, of the British Museum, has a similar one in his collection, given to him by Sir Joseph Banks. The remarkable appearances in this skull are, the extreme smallness and flatness of the os frontis, with a considerable spherical protuberance of the parietal bones, and irregular projections of the lower part of the occipital. Perhaps we shall explain its characters more clearly to some of our readers, by saying that Gall would say, if he still cherished his craniological, or, as we must term them, it seems, his phrenological, reveries, there are signs of but very imperfect development of the organs of the faculties of *phénomènes*, *causalité*, *comparaison*, and *bienveillance;* and great development of those of *circonspection*, *penchant à cacher (secretivité)*, *amativité*, and *philogeniture.*

The manner in which the etchings in this fasciculus are executed, leads us to expect that a series of similar illustrations of the diseases of the bones, in effecting which Mr. Wadd has been for some time past engaged, will prove of much value: this is, indeed, the part of pathology in which the aid of the graphic art can be most usefully and accurately employed.

---

***Observations on Strictures of the Rectum, and other Affections which diminish the Capacity of that Intestine; including Spasmodic Constriction of the Anus, the Hæmorrhoidal Tumors, (called Piles,) Excrescences, and the Prolapsus Ani; and the Mode of Treatment; accompanied with Cases and Engravings.*** By W. White, Member of the Royal College of Surgeons, London; Corresponding Member of the London Medical Society; and one of the Surgeons to the City Infirmary and Dispensary, Bath. Third Edition, improved. pp. 172. H. Gye, Bath; Longman and Co. London. 1820.

It is a curious circumstance in the history of medical opinions, that several surgeons, well informed on the subjects of their art in general, still doubt of the existence of simple permanent stricture of the rectum; that is, a permanent constriction of some part of the rectum, unattended with any sensible morbid change of the structure of the contracted part. What is called schirro-contracted rectum, they acknowledge to be of not unfrequent occurrence; and this they consider to be identical in nature with the disease first mentioned, differing from it only in degree and duration. This was, indeed, the original opinion of Mr. White; but the very extensive experience he has more lately had in the affections under consideration, has led him to consider them as essentially different; and it is with the intention of pointing out the results of his observations respecting the origin of simple stricture of the rectum, that we take up the present edition of his work. We shall not discuss the disputed point above alluded to, because we have no more doubt of the existence of simple stricture of the rectum, than we have of a similar affection of the urethra. We have very lately seen a well-marked case of it, in which the treatment recommended by Boyer* produced an immediate cure. We were induced to resort to this method, in consequence of the inability of the patient to bear the use of bougies or tents, or any distensive force, on the constricted part, however gradually employed.

---

* For an account of this, see the Number of this Journal for January 1819.

Rigors and a febrile paroxysm were the constant results of such measures. Mr. White, when speaking of Boyer's practice, says,

"With respect to the division of the sphincter by the bistoury, the method practised by Mons. Boyer, I am inclined to think that, whether the disease occurs as a primary or secondary affection, the bougie will be always found sufficient to overcome the spasmodic action, when properly employed. If however the bougie should fail in producing a relaxation of the sphincter, I would certainly have recourse to Mons. Boyer's method; having contemplated performing that operation, in the event of the other means failing, long before I met with that gentleman's mode, announced in the London Medical and Physical Journal. But I think every candid practitioner will agree with the sentiment, that, if equal benefit can be obtained by a method which does not derange the structure of the part, it ought to be preferred."

Mr. White adds, in a note,

"I am so truly sensible of the extreme morbid irritability of the part, and of the acute pain introducing the bougie occasions, that nothing short of the fullest confidence in the means proving successful, would embolden the practitioner to persevere, or induce the patient to submit: nevertheless, severe as it is, I think it a better plan than to divide the sphincter, especially should the complaint be attended with a stricture higher up the passage, which cannot be removed by that operation."

We think that cases of the kind above alluded to, are not of very unfrequent occurrence amongst those which affect persons who have long lived in hot climates, as was the case with our patient; and, in this instance, we have but little doubt that the irritation of a bougie would, if the use of this instrument had been long continued, have destroyed the patient. The rigors became each time more severe, instead of diminishing. We must however add, that he bore Desault's bougie, which is particularly recommended by Mr. White, tolerably well at the end of a few days after the operation; and it was then employed with a view to render the cure permanent and complete.

We shall transcribe Mr. White's observations on what he conceives to be the most common origins of this disease.

"Although it would be absurd to suppose, that every case of habitual costiveness proceeded from a mechanical obstruction of the passage, yet, from various conversations I have had with different sensible persons (some medical), who laboured under strictures of the rectum, I am much inclined to think, that the most frequent predisposing cause, is the gut being somewhat narrower about the termination of the sigmoid flexure of the colon than it ought to be, for the purpose of allowing a free and easy passage to the fæces. I was led to this opinion, in consequence of patients having so often stated to me, that, so long as they could remember, they never had a natural motion without experiencing more or less difficulty. It will then appear obvious, that, if the passage should be preternaturally small, it must necessarily form an impediment to the free discharge of the fæces, and thus a foundation will be laid for a greater degree of contraction, as reiterated pressure from the accumulation of fæces must tend to induce a spasmodic action of the intestine; and this, for a length of time repeated, may ultimately produce a more permanent state of contraction, increasing in proportion as the part becomes more deranged in its organization.

"In stating what I conceive to be the most frequent predisposing cause of strictures in the rectum, I do not mean to exclude other circumstances which may contribute to produce the disease: for it is evident, the intestinal canal must be liable to have its peristaltic motion deranged from various causes, such as acrid substances taken into the stomach, a morbid state of the secretions, more especially the biliary; which, by inducing an increased vascularity of the mucous membrane of the intestine, may prove an exciting cause of stricture, agreeable to the observation of that eminent and distinguished physician, Dr. Parry. 'In many cases of dyspepsia,' he says, 'the primary disorder exists in the colon, the villous coat of which appears to be affected with morbid sensibility, unspeakable uneasiness, burning heat, and all those other circumstances which have been described as occurring in the stomach. This state is very apt to run into inflammation, and is, I believe, a frequent origin of strictures in the intestine.' Though I perfectly agree with Dr. Parry, that an inflamed state of the mucous membrane may sometimes prove an exciting cause of stricture, by inducing a spasmodic action of the muscular coat of the intestine; yet I am convinced, from attentive observation, that a disordered state of the colon, very similar to what he describes, is frequently the effect of stricture near the termination of the colon, or in the rectum. The ceasing of these symptoms, when the mechanical obstruction is overcome, affords the strongest proof that the opinion is well founded; this fact having been witnessed in a variety of cases. As Dr. Parry very justly remarks, the secondary affection is often mistaken for the primary, and this I apprehend to be another reason why strictures of the rectum are so frequently overlooked; for it often happens that the patient suffers more pain and uneasiness from a deranged state of the colon, as a consequence of stricture, than from the stricture itself; which is occasioned by the colon being repeatedly distended from accumulation of feculent matter, owing to the difficulty of the fæces passing through a part naturally too narrow, or become so by the formation of a stricture. Hence the colon is weakened, rendered irritable and spasmodic, and, from repeated distension, it becomes greatly enlarged in its capacity."*

Mr. White, in another part of the present work, points out another circumstance which he believes has a considerable ten-

* In a case related by Dr. R. White, he observes, that the whole of this gut was enormously distended, measuring not less in any part than twelve inches in circumference.

dency to the production of this disease, and we fully concur with him in this respect,—which is, the restraint often imposed by our social habits in submitting to the incitements to evacuate the fæces.

We should add the following observations on the situation of the disease, as of great importance in the treatment; because, it must be evident, it is only when the stricture exists near the anus, that the practice of Boyer can be resorted to. Perhaps it will be found, that it is especially the stricture in the situation just designated which is so very intolerant of the agency of the bougie in some cases.

"The situation in which we meet with strictures in the alimentary canal, is most commonly about the termination of the colon. This may be reasonably expected, when we take into consideration that the gut is naturally more exposed to pressure at its curvature, (where its diameter is generally least,) and at the projection of the sacrum,* from the accumulation and passage of hardened fæces, than any other part of the canal. Although I have just stated that, when a stricture is discovered in this situation, there is often another a few inches lower in the gut, yet, I must beg leave to observe, this does not uniformly happen, having met with several cases of stricture about the termination of the colon, where there has been none lower in the intestine: and sometimes strictures have been found between three and four inches from the anus, where there has been none higher. I once met with a semicircular contraction, about two inches from the anus, which occupied the posterior portion of the gut: perhaps, in the course of time, it might have extended entirely round the intestine."

The author has added some new cases to the present edition of this work, that more clearly illustrate the nature of the disease under express consideration, as well as the method of cure, than some of those in the former editions, now omitted. These add much to its value; for there is no disease to which the maxim *Difficile iter per precepta; breve et efficax per exempla*, is more correctly applicable than to that here alluded to. The reputation Mr. White has acquired, has given him such extensive practice in the treatment of this affection, and it is one of so serious a nature, that we think it culpable for any surgeon to neglect to make himself acquainted with the results of his experience. With such sentiments, we recommend it to the perusal of our readers.

* Although sometimes the projection is very considerable, yet I cannot conceive how that can possibly be mistaken for a stricture (as asserted by Mr. Copeland) by any person who is at all acquainted with the anatomy of the part, or in the habit of employing a bougie; as the sensation occasioned by the resistance is so very different.

